# Gut microbiota and metabolome signatures in preterm infants with high versus low risk for neurodevelopmental impairment: a prospective, matched, longitudinal multi-omics study

**DOI:** 10.3389/fcimb.2026.1799859

**Published:** 2026-04-07

**Authors:** Yan-ping Tian, Qing-hong Li, Yi-ming Li, Jia-yuan Zhao, Xin-xin Wei, Jin-ying Wang, Yu-lan Zhou, Shun-bo Yang, Wei Li, Pi Guo, Ling-xi Wang, Ting-ting Dai, Su-fen Hu, Zeng-quan Zhong, Ying-mei Xie, Zhi-hai Lv

**Affiliations:** 1 Department of Rehabilitation Medicine (Pediatric Rehabilitation Division), Longgang District Maternity & Child Healthcare Hospital of Shenzhen City (Longgang Maternity and Child Clinical College of Shantou University Medical College), Shenzhen, China; 2 Department of Preventive Medicine, Shantou University Medical College, Shantou, China; 3 Department of Breast and Thyroid Surgery, First Affiliated Hospital of Army Medical University, Chongqing, China; 4 Department of Science and Education, People's Hospital of Shantou, Shantou, China; 5 Longgang Maternity and Child Institute of Shantou University Medical College (Longgang District Maternity & Child Healthcare Hospital of Shenzhen City), Medical Research Institute of Maternal and Child, Shenzhen, China

**Keywords:** Akkermansia muciniphila, biomarker, dysbiosis, Klebsiella variicola, meconium, metabolomics, microbiota-gut-brain axis, multi-omics

## Abstract

Preterm birth is a leading global cause of neurodevelopmental impairment (NDI), yet early predictive biomarkers remain elusive. The gut microbiome, developing in parallel with the brain and communicating via the microbiota-gut-brain axis, holds potential as a source of such biomarkers. However, specific longitudinal multi-omics signatures predictive of NDI risk in preterm infants are poorly defined. We conducted a prospective, matched, longitudinal study of 60 preterm infants, classified at 3 months corrected age (CA) into high-risk (HR, n=30) or low-risk (LR, n=30) groups for NDI based on combined motor (TIMP) and neurological (GMs) assessments. Fecal samples from birth (meconium) and 3 months CA underwent shotgun metagenomic sequencing and untargeted metabolomics. Groups were rigorously matched for gestational age, birth weight, sex, and clinical exposures. While α- and β-diversity did not differ between groups, profound taxonomic and functional divergence emerged. At 3 months CA, the LR gut was enriched with *Akkermansia muciniphila*, whereas the HR gut was dominated by *Klebsiella variicola*. Functional metagenomics revealed a dysbiotic HR trajectory, enriching pathways for bacterial virulence, stress response, and—notably—multiple pathways annotated for human neurodegenerative diseases, contrasting with LR expansion of core biosynthesis. Metabolomics confirmed a dysfunctional HR state, showing impaired amino acid metabolism and aberrant neuroactive pathway enrichment. Critically, meconium features correlated with 3-month neurobehavioral scores, demonstrating ultra-early predictive potential. Integrated networks at 3 months directly linked *Akkermansia muciniphila* and co-varying glycerophospholipids to superior neurodevelopmental scores, forming a beneficial “*Akkermansia*-lipid” axis, while *Klebsiella variicola* and triterpenoids formed a dysbiotic hub. Our study defines a high-risk gut ecosystem trajectory in preterm infants, characterized by early commensal depletion, pathobiont expansion, and a functional shift towards inflammation and neuroinflammation. These signatures offer novel targets for early risk prediction and microbiome-targeted interventions.

## 1 Introduction

Preterm birth, defined as delivery before 37 weeks of gestation, remains a leading global cause of childhood mortality and long-term neurodevelopmental impairment (NDI), encompassing cerebral palsy, cognitive deficits, learning disabilities, and behavioral disorders (Blencowe et al., 2012). The early postnatal period is now recognized as a critical window of parallel and interconnected development for both the brain and the gut microbiome (Hill et al., 2017; Thompson et al., 2015). Microbes colonize the human body during the first moments of life and coexist with the host, with the intestinal microbiota and their metabolites playing a crucial role in programming important bodily systems such as the immune and central nervous systems during these formative windows, with lifelong structural and functional implications (Cowan et al., 2020). Consequently, perturbations of the developing gut microbiota in early life can have a lasting impact on neurodevelopment and potentially lead to NDI later in life (Borre et al., 2014).

The gut microbiome of preterm infants is distinct and vulnerable. Shaped by clinical necessities such as antibiotic exposure, parenteral nutrition, and delayed enteral feeding, it is often characterized by low diversity, dominance of facultative anaerobes, and a paucity of beneficial symbionts (Gasparrini et al., 2019). A growing body of evidence links specific gut microbial features in preterm infants to later neurobehavioral outcomes (Masi and Stewart, 2019) This is grounded in the well-established concept of the microbiota-gut-brain axis, a bidirectional communication network highlighted by extensive preclinical and clinical research (Morais et al., 2021; Cryan et al., 2019). The early postnatal period represents a critical window where the parallel development of the brain and gut microbiota occurs, leading to the hypothesis of “nested sensitive periods” wherein their interaction shapes future neurological and behavioral outcomes (Carlson et al., 2018). The gut microbiome influences the central nervous system through multiple pathways, including the production of neuroactive metabolites (e.g., short-chain fatty acids, serotonin precursors), regulation of immune system maturation, and modulation of blood-brain barrier integrity (Silva et al., 2020).

Compared to term infants, the developing microbiome of preterm infants is often characterized by reduced diversity, delayed colonization by beneficial symbionts such as *Bifidobacterium*, and a predominance of facultative anaerobes and potential pathobionts like *Enterobacteriaceae* and *Klebsiella* (Gibson et al., 2016; Gasparrini et al., 2019). This aberrant microbial succession is likely driven by frequent exposures in the neonatal intensive care unit (NICU), including broad-spectrum antibiotics, varying nutritional sources, and a controlled environment (Yee et al., 2019; Bokulich et al., 2016). While pioneering studies have begun to associate specific gut microbial features with neurodevelopmental outcomes in preterm infants (Pammi et al., 2017; Masi and Stewart, 2019), most have been limited by small sample sizes, a focus on later-age outcomes, or a lack of integrative multi-omics data. Consequently, the specific longitudinal gut microbial and metabolic signatures that differentiate preterm infants at high risk for NDI early in life remain poorly defined.

Therefore, a critical gap persists. We lack a comprehensive, longitudinal understanding of how the functional potential and metabolic output of the preterm gut ecosystem differentiate infants on high- versus low-risk neurodevelopmental paths from the earliest moments of life. We hypothesize that preterm infants at high risk for NDI harbor distinct gut microbiome and metabolome profiles that are evident from birth (in meconium) and become increasingly pronounced by 3 months corrected age (CA), prior to the manifestation of overt clinical symptoms. To test this, we conducted a prospective, longitudinal, matched-cohort study employing integrated shotgun metagenomics and untargeted metabolomics. Our aims were to: (i) define the taxonomic and functional trajectories of the gut microbiome associated with neurodevelopmental risk; (ii) characterize the corresponding fecal metabolomic profiles; (iii) integrate multi-omics data to construct microbe-metabolite interaction networks; and (iv) directly correlate these gut ecosystem features with standardized neurobehavioral assessments at 3 months CA.

## 2 Methods

### 2.1 Study design and participant enrollment

This prospective longitudinal cohort study was conducted in the Level III/IV Neonatal Intensive Care Unit (NICU) at Longgang District Maternity & Child Healthcare Hospital of Shenzhen City. The primary objective was to compare gut microbiome characteristics between risk groups at birth and at 3 months CA. For sample size estimation, we selected alpha diversity as the primary outcome measure, as it represents a global index of microbial community maturity. Based on an anticipated moderate-to−large effect size (Cohen’s d = 0.75) for alpha diversity differences, informed by previous preterm infant microbiome study (Gibson et al., 2016), a sample size of 29 infants per group was calculated to achieve 80% power at a two-sided α level of 0.05. Our final cohort of 30 matched pairs (60 infants) exceeds this requirement, providing adequate statistical power to detect meaningful differences in microbial community characteristics between the risk groups, as well as to support taxonomic and functional analyses. Between February 1, 2024, and September 30, 2025, we screened 106 infants born at <37 weeks of gestational age for eligibility. Exclusion criteria were: (1) major congenital malformations (e.g., complex congenital heart disease, gastroschisis, neural tube defects); (2) known genetic or chromosomal syndromes; (3) congenital infections (TORCH); and (4) severe perinatal asphyxia (defined as Apgar score <3 at 5 minutes or umbilical cord pH <7.00). Written informed consent was obtained from the parents or legal guardians of all participants prior to enrollment. This study was approved by the Ethics Committee of Longgang District Maternity & Child Healthcare Hospital of Shenzhen City (Approval No: LGFYKYXMLL-2024-01). All procedures were performed in accordance with the Declaration of Helsinki.

### 2.2 Neurodevelopmental risk stratification protocol

The primary outcome for group stratification was neurodevelopmental risk status at 3 months CA, determined using a two-tiered, standardized assessment performed by certified assessors blinded to all clinical and microbiome data.

The 3-month assessment, while early, was chosen based on established literature demonstrating the predictive validity of these tools for later neurodevelopment (Hadders-Algra et al., 1997; Einspieler and Prechtl, 2005). We acknowledge that this is an early endpoint and have planned subsequent follow-up assessments at 12 and 24 months CA to confirm long-term outcomes.

#### 2.2.1 Test of infant motor performance

The TIMP is a validated, norm-referenced assessment of postural and selective motor control for infants from 34 weeks post-conceptional age to 4 months post-term (Campbell et al., 2002, 1995). It evaluates both observed and elicited movements. The total raw score was converted to a percentile rank based on published normative data. A score below the 25th percentile was classified as indicative of impaired motor performance.

#### 2.2.2 General movements assessment

The GMs assessment is a qualitative, video-based evaluation of spontaneous movement patterns that is a highly sensitive and specific predictor of later cerebral palsy (Seesahai et al., 2021). At the 3-month CA time point (the “fidgety movements” period), the presence of normal fidgety movements is the key marker of neurological integrity. Their absence is classified as “definitely abnormal” GMs.

### 2.3 Stratification and matching criteria

#### 2.3.1 High-risk group

Infants presenting with both (a) a TIMP score <25th percentile and (b) definitely abnormal GMs (absent fidgety movements).

#### 2.3.2 Low-risk group

Infants presenting with both (a) a TIMP score ≥25th percentile and (b) normal fidgety GMs.

Infants with discordant results (e.g., abnormal TIMP but normal GMs, or vice versa) were excluded from the primary comparative analysis to ensure a clear phenotypic contrast between risk groups.

To isolate the effect of neurodevelopmental risk from powerful confounders of the early gut microbiome, we employed a rigorous 1:1 matched case-control design after stratification. Each infant in the HR group was manually paired with an infant from the LR pool based on the following matching variables, in hierarchical order: 1) gestational age at birth (± 1 week); 2) Sex (exact match); 3) Birth weight (± 100 g); and 4) primary feeding mode at the time of the 3-month sample collection (exclusive human milk, exclusive formula, or mixed feeding). This process yielded the final, well-phenotyped analytical cohort of 30 matched pairs (n = 60 infants: 30 HR, 30 LR).

### 2.4 Sample collection, processing, and storage

Fecal samples were collected at two pre-defined time points: TP1 (Birth): The first passed meconium stool was collected within 24–72 hours after birth using sterile collection kits. TP2 (3 months CA): A fecal sample was collected at the routine 3-month CA follow-up visit. All samples were immediately placed on ice, transported to the laboratory within 2 hours, aliquoted into sterile cryovials, and stored at −80 °C until subsequent DNA and metabolite extraction. Stringent contamination control protocols were followed throughout.

### 2.5 Shotgun metagenomic sequencing and bioinformatics analysis

#### 2.5.1 DNA extraction and library preparation

Microbial genomic DNA was extracted from approximately 200 mg of fecal homogenate using the QIAamp PowerFecal Pro DNA Kit (Qiagen), which includes mechanical bead-beating to ensure lysis of Gram-positive bacteria. DNA quality and concentration were assessed via fluorometry (Qubit) and gel electrophoresis. Sequencing libraries were prepared using the Illumina DNA Prep kit and sequenced on an Illumina NovaSeq 6000 platform (2 × 150 bp paired-end reads), generating a minimum of 10 million high-quality reads per sample.

#### 2.5.2 Bioinformatic processing

Raw reads were processed through a standardized pipeline:

1. Quality Control and Host Depletion: Reads were trimmed for adapters and low-quality bases using Trimmomatic v0.39. Reads aligning to the human reference genome (hg38) were removed using KneadData (with Bowtie2).
2. Taxonomic Profiling: Species-level relative abundance was determined using MetaPhlAn4, which leverages a database of clade-specific marker genes.
3. Functional Profiling: Microbial community functional potential was analyzed with HUMAnN3, which maps reads to the UniRef90 protein database and subsequently to KEGG Orthology terms and pathways.
4. Diversity Analysis: Alpha-diversity (Observed Species, Shannon Index) and beta-diversity (Bray-Curtis dissimilarity matrix) were calculated from the MetaPhlAn4 output using the vegan package in R. Principal Coordinate Analysis (PCoA) was used for beta-diversity visualization.

### 2.6 Untargeted metabolomic profiling by LC-MS

#### 2.6.1 Metabolite extraction

Metabolites were extracted from approximately 50 mg of fecal sample using a cold methanol:water (80:20, v/v) solution containing internal standards for quality control. Samples were vortexed, sonicated, and centrifuged. The supernatant was dried under a gentle stream of nitrogen and reconstituted in an appropriate injection solvent.

#### 2.6.2 LC-MS analysis

Metabolic profiling was performed on a Thermo Scientific Q Exactive HF-X hybrid quadrupole-Orbitrap mass spectrometer coupled to a Vanquish Horizon UHPLC system. Chromatographic separation was achieved on a SeQuant ZIC-pHILIC column (150 × 2.1 mm, 5 μm) under HILIC conditions. The mass spectrometer operated in both positive and negative electrospray ionization modes with full-scan MS (m/z 70–1050) at a resolution of 120, 000. Pooled quality control samples were analyzed throughout the sequence to monitor instrument stability.

#### 2.6.3 Metabolomics data processing

Raw data files were converted to mzML format and processed using the XCMS package in R for peak picking, alignment, and retention time correction. Metabolites were annotated by matching accurate mass (± 5 ppm) and, when available, MS/MS fragmentation spectra against public databases (HMDB, METLIN, LipidMaps) and an in-house spectral library. Identification confidence was reported according to the Metabolomics Standards Initiative levels.

### 2.7 Data availability

The raw metagenomic sequencing data supporting the findings of this study have been deposited in the NCBI Sequence Read Archive (SRA) under BioProject accession number PRJNA1428929. The raw metabolomics data have been deposited in the MetaboLights database under accession number MTBLS13962. Both datasets will be made publicly available upon publication of this manuscript.

### 2.8 Statistical analysis

#### 2.8.1 Clinical and demographic data

Continuous variables were summarized as mean ± standard deviation or median [interquartile range] based on distribution and compared between matched HR and LR groups using the paired Wilcoxon signed-rank test or the Mann-Whitney U test, as appropriate. Categorical variables were presented as counts (percentages) and compared using McNemar’s test or Fisher’s exact test. A two-sided p-value < 0.05 was considered statistically significant.

#### 2.8.2 Microbiome and metabolome data analysis

The primary analysis for identifying features associated with neurodevelopmental risk was performed using MaAsLin2 (Multivariate Association with Linear Models 2). MaAsLin2 applies linear (or generalized linear) models to identify associations while adjusting for specified covariates. For all models, we adjusted for the following *a priori* selected confounders: delivery mode (vaginal/cesarean), birth weight (continuous), sex, gestational age (continuous), and a binary indicator for culture-proven neonatal infection. For metabolomics data, normalization was performed using the total ion current. Exploratory analysis for differentially abundant taxa was also conducted using Linear Discriminant Analysis Effect Size (LEfSe), with an LDA score > 2.0 and p < 0.01. To account for multiple testing, the Benjamini-Hochberg procedure was applied to all statistical tests (including MaAsLin2, LEfSe, and correlation analyses) to control the false discovery rate (FDR). An FDR-adjusted q-value < 0.10 was set as the threshold for significant discovery, with features meeting q < 0.05 highlighted as high-confidence findings.

#### 2.8.3 Network and integration analysis

Spearman rank correlation coefficients were calculated between significantly differentially abundant microbial species and metabolites. Mantel tests were performed to assess the overall correlation between microbial (Bray–Curtis) and metabolic (Euclidean) distance matrices. For network visualization, we retained correlations with |r| ≥ 0.25, as this threshold was empirically determined to balance the inclusion of biologically plausible, moderate-strength associations while reducing visual noise from very weak correlations. All reported correlations in the network also met a nominal p-value < 0.05. Correlation networks were visualized using Cytoscape v3.9.1. For integration with neurodevelopmental scores, tripartite networks were constructed incorporating key microbes, metabolites, and scores from the Alberta Infant Motor Scale (AIMS) and Griffiths Mental Development Scales.

## 3 Results

### 3.1 Cohort characteristics and efficacy of the matched design

**Figure 1:**
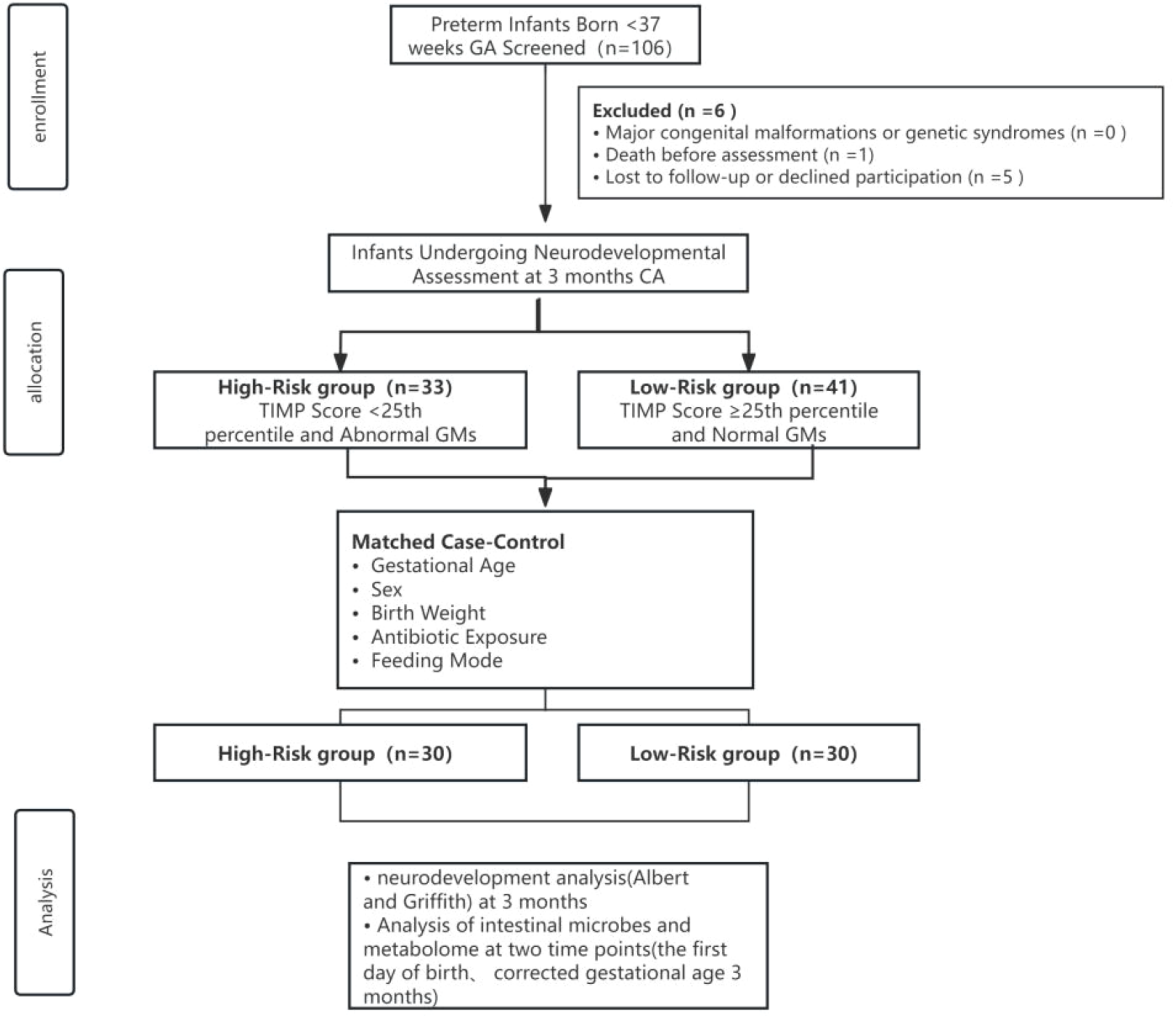
Study flowchart.

From the 106 infants screened, 100 completed the 3-month CA neurodevelopmental assessment. Application of the strict, dual-assessment risk stratification criteria identified 33 HR and 41 LR infants. After implementing the 1:1 matching protocol, 30 perfectly matched pairs were formed, constituting the final analytical cohort of 60 infants (**Figure 1**: Study Flowchart). The success of the matching procedure is demonstrated in **Table 1**. The HR and LR groups were statistically indistinguishable across all major perinatal and early clinical parameters that are known to significantly influence the early gut microbiome. Crucially, there were no significant differences in gestational age, birth weight, sex distribution, mode of delivery, exposure to intrapartum antibiotics, incidence of bronchopulmonary dysplasia, or feeding mode at the time of the 3-month sampling. This rigorous matching effectively minimizes the likelihood that subsequent multi-omics differences are driven by these extrinsic clinical factors, thereby strengthening the inference that observed disparities are more directly linked to the neurodevelopmental risk phenotype itself.

**Table 1:** Baseline characteristics of low-risk versus high-risk preterm infants.

Variable	Low-risk group	High-risk group	P*-*value
GA at birth, weeks (median [SD])	33.63 ± 2.31	33.83 ± 2.41	0.744
Female infants (%)	15 (50)	11 (36.67)	0.297
Birthweight, g (mean [SD])	2162 ± 469.49	2132 ± 578.57	0.826
Vaginal delivery (%)	14 (46.67)	9 (30)	0.184
Labor antibiotics (%)	2 (6.67)	3 (10)	0.64
Bronchopulmonary dysplasia (%)	12 (40)	9 (30)	0.417
Breastmilk exposure (%)	28 (93.33)	25 (83.33)	0.306
Neonatal Jaundice (%)	18 (60)	19 (63.33)	0.791

### 3.2 Postnatal maturation is the primary driver of overall microbial community structure, obscuring risk-related differences

**Figure 2:**
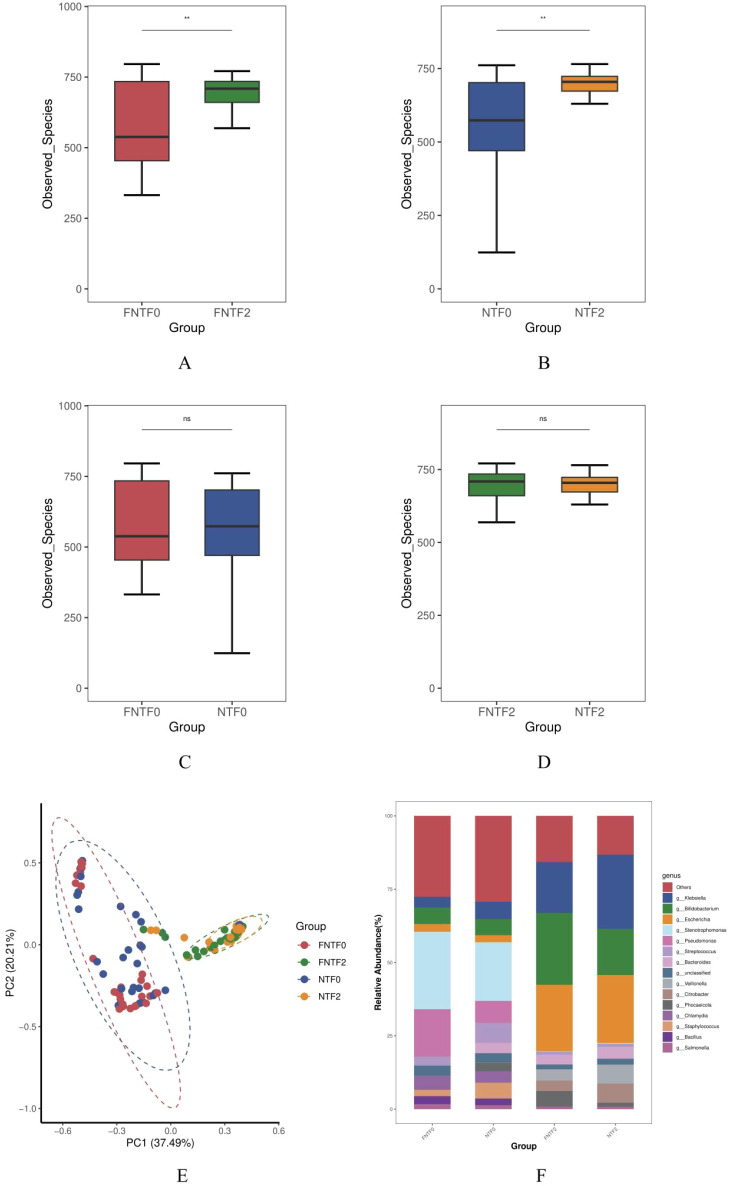
Gut microbial community structure and development in preterm infants stratified by neurodevelopmental risk. FNTF0, low-risk group at meconium; FNTF2, low-risk group at 3 months CA; NTF0, high-risk group at meconium; NTF2, high-risk group at 3 months CA. **(A, B)** Alpha-diversity (Observed Species) within the low-risk (LR, A) and high-risk (HR, B) groups from birth (meconium, TP1) to 3 months CA (TP2). Box plots show median and interquartile range; p-values from paired tests. **(C, D)** Species richness comparison between risk groups **(C)** at birth and **(D)** at 3 months CA. Box plots show median and IQR; ns, not significant (Mann–Whitney U test). **(E)** Principal Coordinate Analysis (PCoA) of Bray–Curtis dissimilarity (family level). Each point represents a sample, colored by time point (TP1: meconium; TP2: 3 months CA) and shaped by risk group (circle: LR; triangle: HR). Axes show percent variance explained. **(F)** Genus-level relative abundance stacked bar plots, grouped by risk status and time point. Only the top 15 most abundant genera across all samples are shown; remaining genera are collapsed into “Other”.

We first examined the macro-scale development of the gut ecosystem. Alpha-diversity increased significantly from meconium (TP1) to 3 months CA (TP2) in both the low-risk (LR) and high-risk (HR) groups (**Figures 2A, B**, p < 0.001 for both, paired Wilcoxon test), indicating expected ecological succession after leaving the NICU environment. Crucially, no significant differences in species richness were observed between the HR and LR groups at either time point (**Figures 2C, D**). Beta-diversity analysis (Principal Coordinate Analysis, PCoA) showed that the largest source of variation separated samples by postnatal age (explaining 37.49% of variance, **Figure 2E**), not by risk group. Genus-level compositional profiles further demonstrated that all infants transitioned from a TP1 microbiota dominated by environmental genera (e.g., *Stenotrophomonas*) to a TP2 microbiota dominated by classic enteric facultative anaerobes (e.g., *Escherichia, Klebsiella*) (**Figure 2F**). These results indicate that the overall timetable and structural assembly of early gut microbial colonization are conserved between HR and LR infants, and neurodevelopmental risk is not associated with a gross failure or significant delay in this ecological succession.

### 3.3 Divergent taxonomic trajectories reveal an early and persistent high-risk pathobiont signature

**Figure 3:**
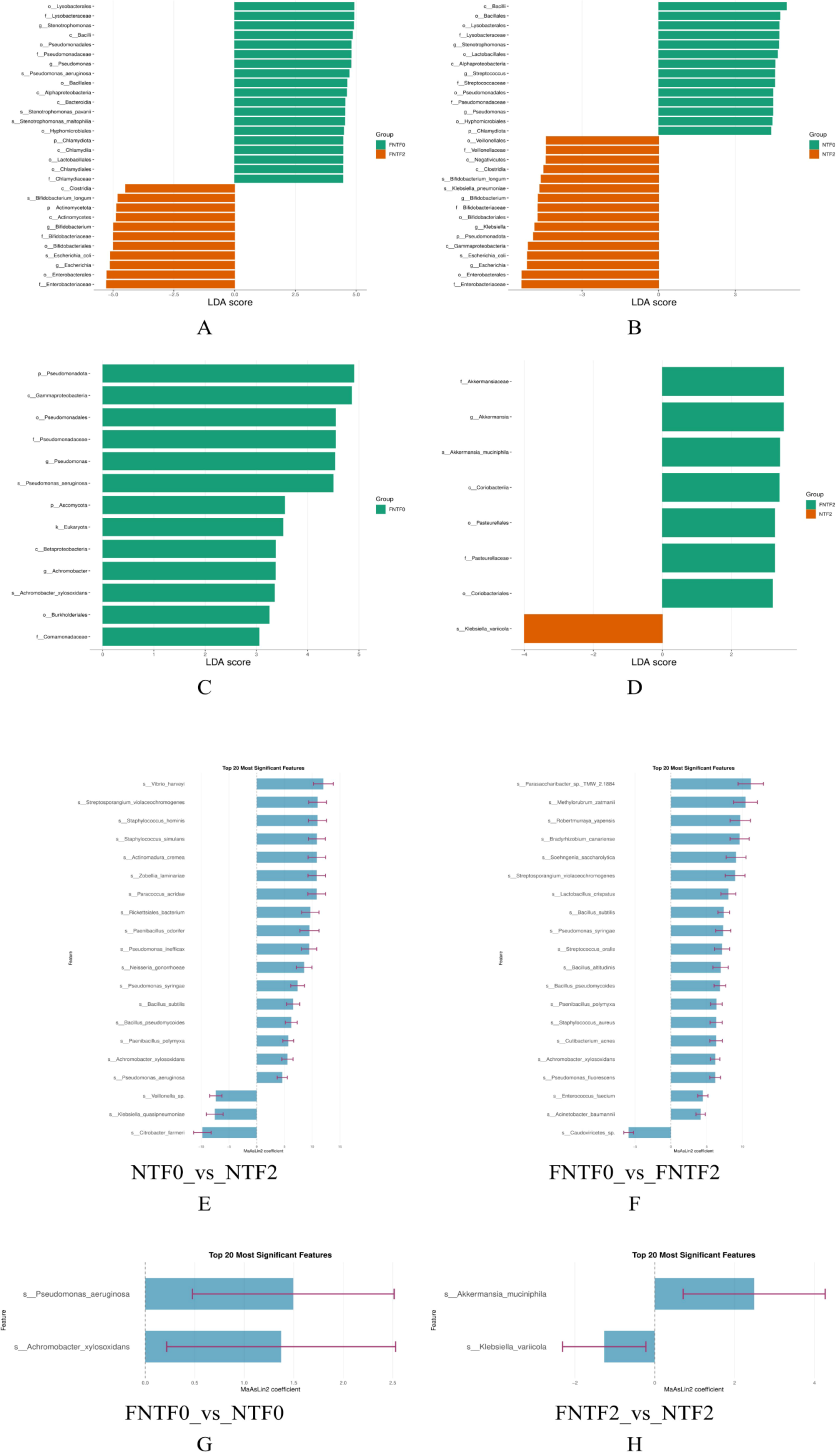
Differential abundance of gut microbial taxa between neurodevelopmental risk groups across time. **(A–D)** Differentially abundant taxa identified by LEfSe analysis. Bar length reflects the Linear Discriminant Analysis (LDA) score (log10). Comparisons shown are: longitudinal change within LR infants (**A**: FNTF0 vs. FNTF2), longitudinal change within HR infants (**B**: NTF0 vs. NTF2), risk comparison at birth (**C**: FNTF0 vs. NTF0), and risk comparison at 3 months CA (**D**: FNTF2 vs. NTF2). **(E–H)** Forest plots showing effect sizes (MaAsLin2 coefficient) and significance for the top 20 species associated with the same four comparisons after covariate adjustment. Key species are highlighted: *Akkermansia muciniphila* (enriched in LR) and *Klebsiella variicola* (enriched in HR).

Beneath this conserved structural framework, we discovered profound differences in bacterial species abundance using covariate-adjusted multivariate association modeling (MaAsLin2) and LEfSe analysis (**Figure 3**). The longitudinal design allowed four key comparisons:

Longitudinal change in LR infants (FNTF0 vs. FNTF2): Development was characterized by an increase in a heterogeneous set of taxa, including environmental species and the commensal *Lactobacillus crispatus* (**Figures 3A, F**), suggesting a resilient and adaptable ecosystem.

Longitudinal change in HR infants (NTF0 vs. NTF2): In stark contrast, the HR trajectory was marked by a significant increase in species linked to environmental reservoirs and opportunistic infections (e.g., *Vibrio harveyi*, *Pseudomonas aeruginosa*), with a notable absence of canonical beneficial commensals like *Bifidobacterium* (**Figures 3B, E**).

Risk comparison at birth (FNTF0 vs. NTF0): The meconium of later HR infants was already enriched with *Pseudomonas aeruginosa* and *Achromobacter xylosoxidans* (**Figures 3C, G**), indicating very early microbial seeding associated with subsequent neurological vulnerability.

Risk comparison at 3 months (FNTF2 vs. NTF2): By this time point, the gut ecosystems had crystallized into a stark opposition. The LR gut was strongly associated with *Akkermansia muciniphila*, while the HR gut was dominated by *Klebsiella variicola* (**Figures 3D, H**). This establishes a clear “*Akkermansia-Klebsiella* axis” as a core taxonomic feature distinguishing neurodevelopmental risk.

### 3.4 Functional metagenomics uncovers a fundamental bifurcation: healthy maturation vs. dysbiotic drift

**Figure 4:**
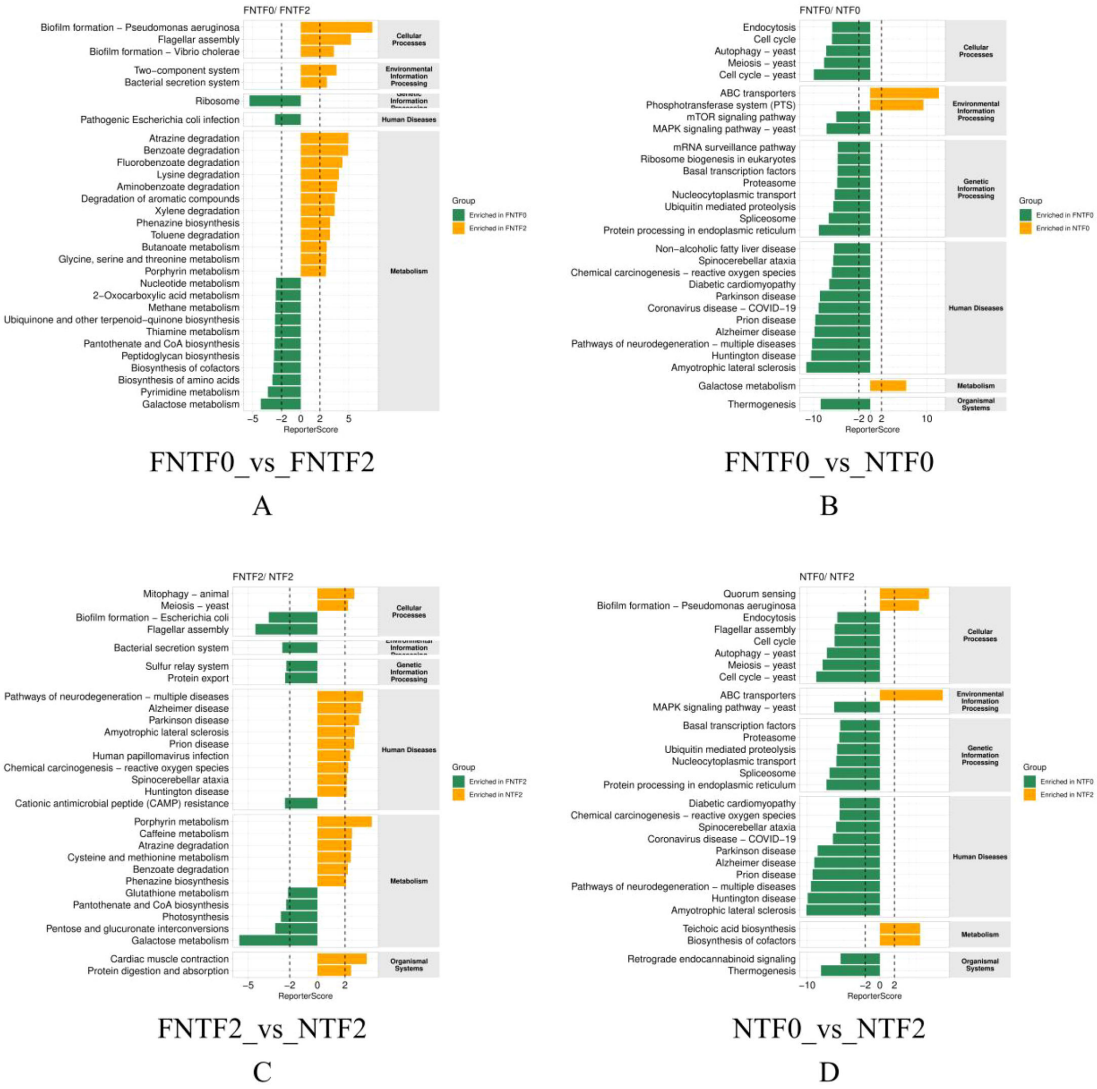
Divergent functional trajectories of the gut microbiome revealed by KEGG pathway analysis. Top 30 enriched pathways (|ReporterScore| > 2, p < 0.05). Bar height: enrichment magnitude; colors: enriched groups. ReporterScore sign: directional (+/−) for pairwise comparisons, non−directional (absolute value) for multi−group comparisons. **(A)** Low-risk infant development (FNTF0 vs. FNTF2). **(B)** Birth comparison (FNTF0 vs. NTF0). **(C)** Risk comparison at 3 months (FNTF2 vs. NTF2). **(D)** High-risk infant development (NTF0 vs. NTF2).

KEGG pathway analysis revealed a profound bifurcation in the functional potential of the microbial communities (**Figure 4**).

LR Functional Trajectory (FNTF0 vs. FNTF2): Exhibited healthy maturation, with significant expansion in core biosynthetic and metabolic housekeeping functions (e.g., amino acid metabolism, vitamin biosynthesis) (**Figure 4A**).

Initial Functional Divergence at Birth (FNTF0 vs NTF0): LR meconium showed enrichment of host-derived eukaryotic pathways and ‘Human Diseases’ pathways (reflecting genes involved in protein folding and oxidative stress), whereas HR meconium was characterized by prokaryotic environmental information processing and metabolic pathways (**Figure 4B**).

Functional Divergence at 3 Months (FNTF2 vs. NTF2): HR microbiota was enriched for pathogenicity-related pathways (flagellar assembly, secretion systems, biofilm formation) and inflammation-associated ‘Human Diseases’ pathways, while LR microbiota showed enrichment of metabolic pathways and host-supportive functions (e.g., protein digestion, amino acid metabolism) (**Figure 4C**).

HR Functional Trajectory (NTF0 vs. NTF2): Followed an aberrant developmental pattern, shifting from host-dominated signals at birth toward enrichment of bacterial stress response and virulence pathways at 3 months (e.g., quorum sensing, biofilm formation, antimicrobial peptide resistance), with sustained enrichment of neurodegeneration-related pathways (**Figure 4D**).It is important to note that these ‘Human Diseases’ pathway annotations—particularly those related to neurodegenerative conditions—are based on orthology mapping and likely reflect the presence of microbial genes involved in core biological modules (e.g., oxidative stress, protein misfolding, inflammatory signaling) that are shared across multiple contexts, rather than implying any disease-specific processes in the infants.

### 3.5 Metabolomic profiling confirms a disrupted gut-brain metabolic state in high-risk infants

**Figure 5:**
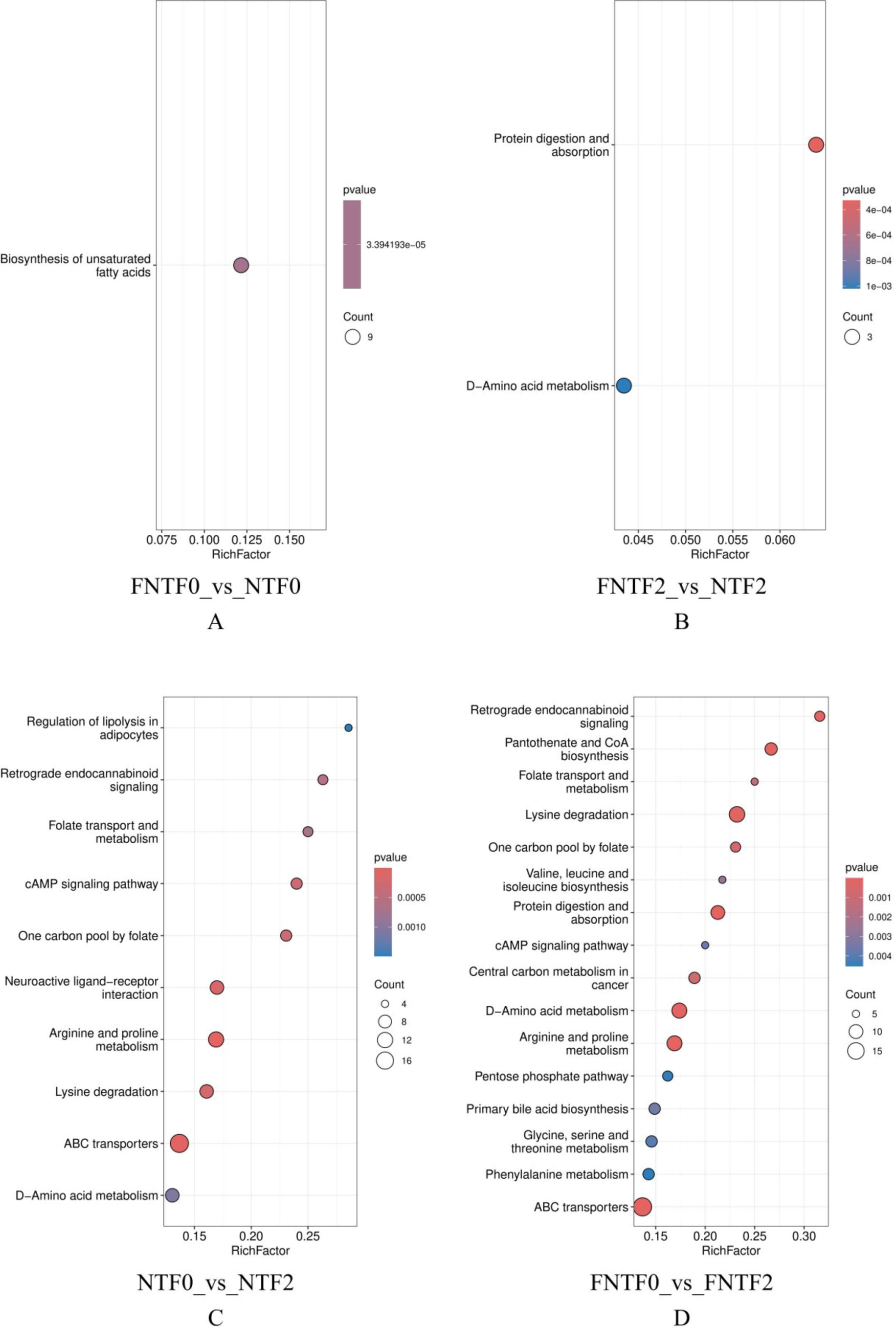
Distinct fecal metabolic profiles between risk groups revealed by KEGG pathway enrichment analysis (top 30 pathways by p-value). Bubble size reflects the number of enriched metabolites; bubble color denotes the enrichment p-value (darker, more significant). **(A)** Birth comparison (FNTF0 vs. NTF0). **(B)** Low-risk group development (FNTF0 vs. FNTF2). **(C)** High-risk group development (NTF0 vs. NTF2). **(D)** Risk comparison at 3 months (FNTF2 vs. NTF2).

Untargeted fecal metabolomics reflected the net output of host and microbial metabolism (**Figure 5**).

Risk Comparison at Birth (FNTF0 vs. NTF0): Differences were minimal (**Figure 5A**).

Risk Comparison at 3 Months (FNTF2 vs. NTF2): The HR metabolome was defined by persistent amino acid metabolic impairment and aberrant neuroactive signaling, including dysregulated “D-Amino acid metabolism” (**Figure 5B**), providing direct evidence that the gut microbiome likely shapes these systemic metabolic disparities.

Longitudinal Change in HR infants (NTF0 vs. NTF2): Development was aberrant, showing concurrent disturbances: (1) impaired core nutrient metabolism (depletion of amino acid pathways) and (2) aberrant neuroactive pathway enrichment (e.g., “Tryptophan metabolism”) (**Figure 5C**).

Longitudinal Change in LR infants (FNTF0 vs. FNTF2): Development indicated coordinated host-microbe collaboration, with enrichment of pathways like “Protein digestion and absorption” and “Endocannabinoid signaling” (**Figure 5D**).

### 3.6 Risk-specific assembly of integrated microbe-metabolite interaction networks reveals ecological disarray

**Figure 6:**
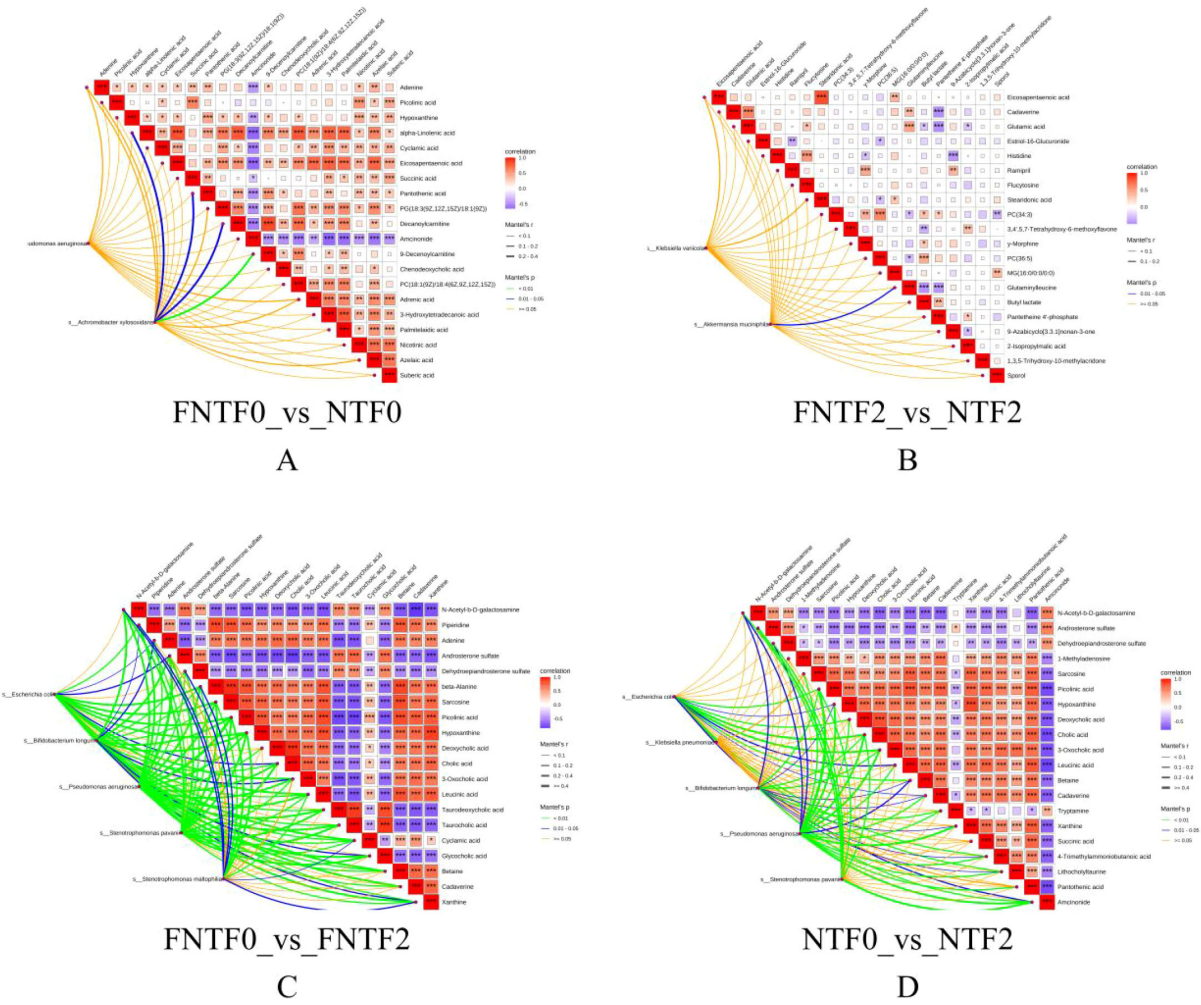
Divergent assembly of gut microbe-metabolite interaction networks between risk groups. Mantel test networks visualize significant Spearman correlations between differentially abundant microbial species and metabolites. Nodes: Yellow circles represent bacterial species, blue squares represent metabolites. Edges: Red lines indicate positive correlations (r > 0), blue lines indicate negative correlations (r < 0); edge thickness scales with the absolute correlation coefficient (|r|). Networks are shown for the birth comparison (**A**: FNTF0 vs NTF0) and the 3-month CA comparison (**B**: FNTF2 vs NTF2). The longitudinal development of these networks is shown for LR (**C**: FNTF0 vs FNTF2) and HR (D: NTF0 vs NTF2) infants. Key interpretation note in results text: At 3 months CA, LR infants establish a dense, cooperative network centered on *Akkermansia muciniphila* linked to beneficial glycerophospholipids (e.g., phosphatidylcholines). In contrast, the HR network remains sparse, lacks a beneficial hub, and is centered on pathobionts like *Klebsiella* and *Escherichia* with persistent negative correlations.

To move beyond individual features and understand the system-level relationships, we analyzed how microbial species and metabolites co-vary to form ecological networks using Mantel tests and correlation analysis (**Figure 6**). This revealed markedly different states of ecological organization.

#### 3.6.1 At birth (FNTF0 vs. NTF0)

The networks were sparse and dominated by negative correlations (blue edges, **Figure 6A**), particularly in HR infants where lower levels of metabolites like decanoylcarnitine (involved in fatty acid metabolism) were associated with higher abundance of *Pseudomonas aeruginosa*. This indicates early, antagonistic host-pathogen or microbe-metabolite dynamics.

#### 3.6.2 At 3 months, low-risk vs. high-risk (FNTF2 vs. NTF2)

A marked ecological shift had occurred (**Figure 6B**). The LR infant network was dense, complex, and dominated by positive, cooperative associations (red edges). *Akkermansia muciniphila* emerged as a central hub, strongly and positively linked to a cluster of beneficial glycerophospholipid metabolites (e.g., phosphatidylcholines PC(34:3), PC(36:4)). This formed a coherent and robust “symbiotic *Akkermansia*-lipid axis.” In stark contrast, the HR network remained sparse, lacked any central beneficial hub, and was characterized by persistent negative correlations. New, concerning pro-inflammatory links emerged, such as a positive correlation between *Escherichia coli* and androgen sulfates, which can have immunomodulatory effects.

#### 3.6.3 Longitudinal trajectory - low-risk (FNTF0 vs. FNTF2)

LR infants successfully transitioned from the sparse, antagonistic birth network to the dense, cooperative 3-month network (**Figure 6C**). This maturation involved the positive integration of taxa like Bifidobacterium longum into associations with bile acid metabolites, indicating developing functional mutualism.

#### 3.6.4 Longitudinal trajectory - high-risk (NTF0 vs. NTF2)

HR infants failed to make this critical ecological transition (**Figure 6D**). Their network remained structurally simple, fragile, and centered on Enterobacteriaceae (*Klebsiella*, *Escherichia*), maintaining inflammatory features and lacking cooperative structure.

In summary, LR infants assemble a robust, metabolically integrated, and cooperative gut ecosystem, resembling a resilient ecological community. HR infants remain in a state of ecological disarray, characterized by antagonistic interactions, a lack of keystone symbionts, and pro-inflammatory host-microbe-metabolite associations. The establishment of the Akkermansia-glycerophospholipid axis appears to be a hallmark of healthy gut ecosystem maturation.

### 3.7 Tripartite networks directly link gut ecosystem features to neurobehavioral performance

**Figure 7:**
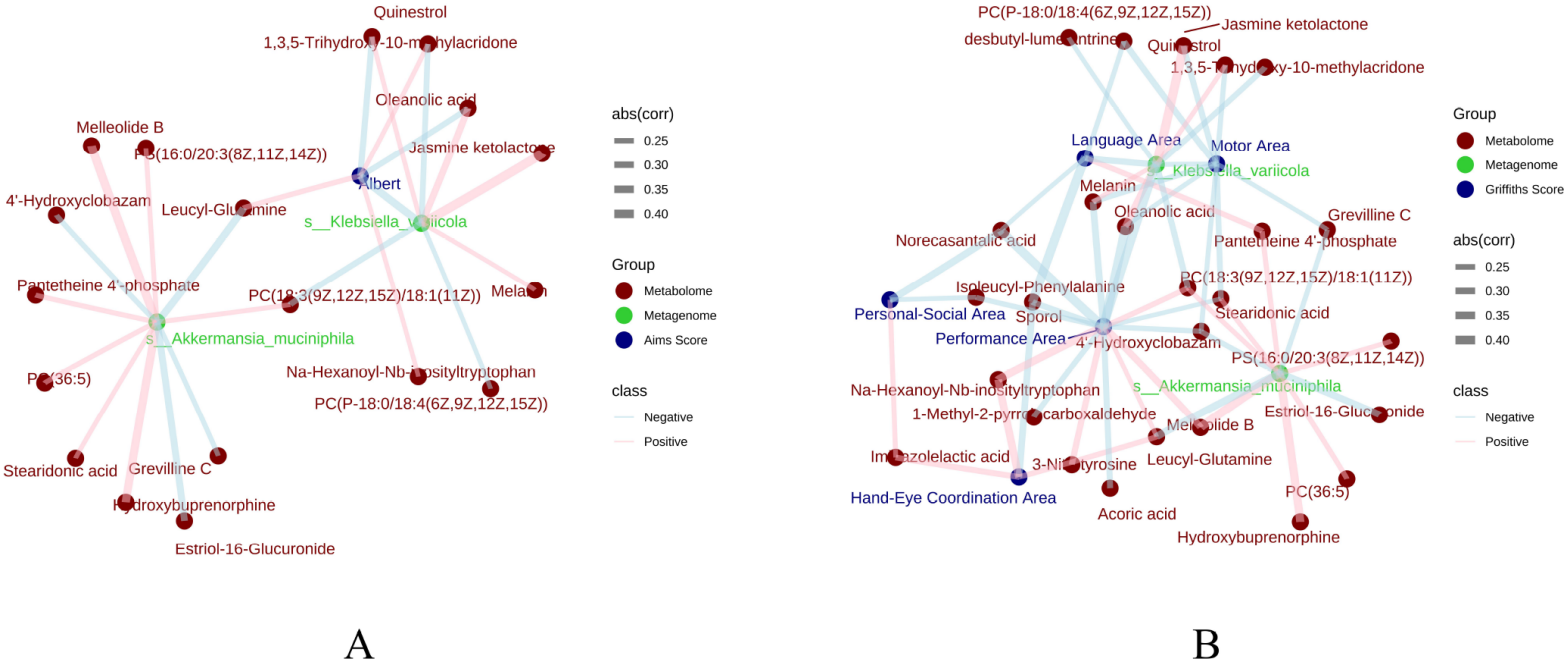
Tripartite networks link gut microbes and metabolites to neurodevelopmental scores. Integrated correlation networks at 3 months CA incorporate key differential bacteria (yellow circles), metabolites (blue squares), and neurodevelopmental scores (green diamonds). Edges represent significant Spearman correlations (|r| ≥ 0.25, p < 0.05), with pink for positive and light blue for negative associations. **(A)** Network centered on the Alberta Infant Motor Scale (AIMS) total score. **(B)** Network incorporating Griffiths Mental Development Scales sub-domains (Language, Motor, Personal-Social, Hand-Eye Coordination, Performance).

Finally, to directly bridge the gap between omics signatures and clinical phenotype, we constructed integrated tripartite correlation networks at 3 months CA (**Figure 7**). These networks incorporated (i) the key differential bacterial species, (ii) the key differential metabolites, and (iii) the neurobehavioral scores (AIMS total and Griffiths sub-domains).

#### 3.7.1 Network centered on the AIMS motor score

The network organized with compelling clarity around two opposing bacterial hubs (**Figure 7A**). *Akkermansia muciniphila* demonstrated a strong positive correlation with the AIMS total score and with a cluster of six glycerophospholipid metabolites. Conversely, *Klebsiella variicola* was negatively correlated with the AIMS score and this same beneficial metabolite cluster. *Klebsiella variicola* was positively associated with a distinct set of metabolites, including triterpenoids like oleanolic acid, defining a coherent “dysbiotic cluster.”

#### 3.7.2 Network with griffiths developmental scores

The pattern was remarkably consistent across multiple developmental domains (**Figure 7B**). In the Griffiths network (**Figure 7B**), multiple phospholipid metabolites—including PC (18:3/18:1) and PS (16:0/20:3)—clustered with developmental scores across all sub-domains, while metabolites such as desbutyl-lumefantrine and oleanolic acid were negatively associated with these scores. Notably, indole-3-lactic acid (labeled as Imazoleacetic acid) appeared in proximity to Hand-Eye Coordination, suggesting a potential neurosupportive role. In contrast, negative correlations were observed with metabolites like drug-derived desbutyl-lumefantrine, which was more abundant in the HR group.

These integrated analyses move beyond simple association to provide a direct, systems-level link between specific gut ecosystem states and tangible neurobehavioral outcomes. They nominate the *Akkermansia muciniphila* glycerophospholipid axis not just as a correlate, but as a compelling, multi-omics biomarker signature for healthy neurodevelopment.

## 4 Discussion

Our prospective, longitudinal, multi-omics study delineates a distinct developmental trajectory of the gut ecosystem in preterm infants who are at high risk for NDI. This trajectory is not defined by a mere delay in microbial colonization or reduced diversity (Yassour et al., 2016), but by an early and persistent functional divergence characterized by the depletion of key commensals, expansion of pathobionts, and a collective shift in microbial community metabolism towards pathways implicated in inflammation and neurotoxicity (Sharon et al., 2016; Valles-Colomer et al., 2019). Critically, signatures of this divergent trajectory are detectable in meconium and correlate with later neurobehavioral performance, suggesting their potential utility as early biomarkers for risk prediction (Grier et al., 2017). We emphasize that these findings demonstrate a strong association, not a proven causal relationship, between this gut ecosystem state and neurodevelopmental outcomes.

The most striking taxonomic finding is the observed dichotomy between *Akkermansia muciniphila* and *Klebsiella variicola. Akkermansia muciniphila*, considered a keystone species for gut health, is associated with enhanced intestinal barrier integrity (Derrien et al., 2017)and modulated host immunity (Ottman et al., 2017), and produces anti-inflammatory metabolites like short-chain fatty acids and amino acid derivatives (Dodd et al., 2017; Cani and de Vos, 2017). *Akkermansia muciniphila* has garnered attention as a “next-generation probiotic” due to its established roles in enhancing intestinal barrier integrity, modulating host immunity, and producing anti-inflammatory metabolites (Derrien et al., 2017; Ottman et al., 2017; Cani and de Vos, 2017). Recent strain-specific safety evaluations have provided important groundwork: a comprehensive assessment of *Akkermansia muciniphila Akk11*, isolated from healthy infant feces, demonstrated no adverse effects in toxicity studies, with no transferable antibiotic resistance genes and excellent gastrointestinal stress tolerance (Wang et al., 2025). These findings support the strain-specific safety profile of *Akkermansia muciniphila* and provide a rationale for further investigation, although causal relationships and optimal intervention parameters for preterm infants require rigorous evaluation in experimental models and preclinical studies before clinical translation can be considered.

Its consistent depletion in the HR group is correlated with poorer neurobehavioral scores. Conversely, the dominance of *Klebsiella variicola*, a member of a genus notorious for nosocomial infections in the NICU (Gorrie et al., 2017), in the HR group is associated with a pro-inflammatory ecosystem state (Martin and Bachman, 2018). This “*Akkermansia-Klebsiella* axis” represents a core ecological association distinguishing the groups, consistent with observations of *Akkermansia muciniphila* depletion in other inflammatory and metabolic conditions (Dao et al., 2016), though its causal role in neurodevelopment remains to be determined.

Beyond taxonomy, our functional metagenomic data provide a insight into the associated community phenotype. The enrichment of pathways related to bacterial virulence (e.g., LPS biosynthesis) and stress response in the HR microbiome indicates a community phenotype geared towards persistence and immune activation (Rooks and Garrett, 2016; Behnsen et al., 2013). LPS is a known trigger for systemic inflammation, a risk factor for white matter injury in preterm infants (Strunk et al., 2014). These pathways contain microbial genes involved in core biological processes—such as oxidative stress and inflammation—that have been associated with neuronal injury in the literature (Xiao, 2024; Wang et al., 2023). It is important to emphasize that this observation reflects microbial gene content related to these fundamental processes, rather than indicating any disease state in the infants. However, it raises the possibility that the functional profile of the dysbiotic HR microbiome overlaps with pathways implicated in neuroinflammation, a factor that may be relevant during this critical developmental window (Bokobza et al., 2019; Drobyshevsky et al., 2024).

The metabolomic findings corroborate and extend this picture of an associated dysfunctional gut-brain metabolic state. The impaired amino acid metabolism and aberrant enrichment of neuroactive pathways (e.g., tryptophan/serotonin) in HR infants suggest a state of metabolic imbalance. The tryptophan-kynurenine pathway, which can be influenced by gut microbes, is known to produce neuroactive metabolites that have been shown to impact brain development in preclinical models (Cervenka et al., 2017).The differential regulation of microbially derived D-amino acids provides compelling circumstantial evidence that the gut microbiome is a key contributor to this metabolic disparity (Sasabe et al., 2016).

The integrated correlation networks strengthen these associations by demonstrating system-level relationships. The identification of *Akkermansia muciniphila* and its co-varying glycerophospholipids as a cohesive “beneficial hub” positively linked to neurodevelopmental scores provides a tangible, multi-omics biomarker signature. Glycerophospholipids are essential for brain development (Hamilton et al., 2007), and their association with a gut symbiont suggests a potential microbiota-dependent pathway for supporting neurodevelopment (Schwarzer et al., 2016). Glycerophospholipids—including phosphatidylcholines, phosphatidylethanolamines, and phosphatidylserines—are fundamental structural components of neuronal membranes and play essential roles in brain development and function (Zhang et al., 2026). As the main lipid constituents of cellular membranes, they are critical for membrane fluidity, vesicle trafficking, and synaptic transmission (Lamari et al., 2025). During perinatal brain development, glycerophospholipids provide the building blocks for rapid synaptogenesis and myelination, with specific fatty acid compositions (particularly long-chain polyunsaturated fatty acids) being preferentially incorporated into developing neural tissues. Studies in animal models of perinatal brain injury have demonstrated that alterations in glycerophospholipid metabolism accompany excitotoxic damage, with decreased levels of specific phosphatidylcholine species observed following neonatal brain lesions (Hermans et al., 2024). Conversely, dietary interventions that increase phospholipid and unsaturated fatty acid levels have been associated with reduced brain damage and improved cognitive outcomes in hypoxic-ischemic encephalopathy models (Chen et al., 2023). The coherence of our multi-omics data—linking a keystone commensal with these neurosupportive lipids and better developmental scores—suggests a potential gut microbiota-dependent pathway for supporting early brain development, though the directionality and causality of these associations remain to be experimentally established. Conversely, the “Klebsiella variicola triterpenoid” hub associated with poorer outcomes defines a clear dysbiotic pattern. Triterpenoids can have complex bioactivities, and their microbial co-association warrants investigation into their role in inflammation or cellular stress (Dzubak et al., 2006). Oleanolic acid is a pentacyclic triterpenoid abundantly present in olive oil and various medicinal plants, with documented pharmacological activities including hepatoprotective, anti-inflammatory, and antioxidant effects. Its co-occurrence with Klebsiella variicola in the high-risk group is noteworthy and invites cautious interpretation. Experimental studies demonstrate its ability to modulate gut microbiota composition and intestinal epithelial immune gene expression (Xue et al., 2022), and protective effects in neonatal contexts have been reported (Fu et al., 2024). The apparent contrast—a molecule associated with protective properties in some settings co-occurring with a dysbiotic, pro-inflammatory ecosystem in our high-risk infants—highlights the critical importance of context in host-microbe-metabolite interactions. A metabolite’s biological effects may be profoundly influenced by the accompanying microbial community, host developmental stage, and physiological milieu (Rooks and Garrett, 2016; Xue et al., 2022). In preterm infants at high neurodevelopmental risk, the presence of oleanolic acid within a *Klebsiella variicola*-dominated ecosystem could reflect context-dependent functionality, altered metabolism by the dysbiotic community, or a host counter-regulatory response. This underscores the need for future studies to investigate metabolite functions within their ecological context and to assess whether the oleanolic acid-neurodevelopment relationship is modified by the surrounding microbial community.

The predictive potential of meconium profiles is a significant finding. The presence of specific bacteria and metabolites at birth in infants later classified as HR indicates that the seeding of the gut ecosystem is associated with future developmental vulnerability. Meconium reflects *in utero* and peripartum exposures (Gosalbes et al., 2013; Chu et al., 2017), suggesting that prenatal factors may set the stage for this divergent trajectory (Arboleya et al., 2015; Gensollen et al., 2016).

Our study has limitations that contextualize the findings. The sample size, though robust for this design, warrants validation in larger, independent cohorts (Knights et al., 2014).Most importantly, the observational, matched-cohort design allows us to control for major confounders and reveal strong associations, but it cannot definitively prove causation. Residual confounding or the possibility that an innate neurodevelopmental vulnerability shapes the gut microbiome (reverse causality) remain plausible. Therefore, our findings should be interpreted as identifying a high-risk biosignature that includes a specific gut ecosystem state.

Future research must address causality. Employing gnotobiotic animal models to test the direct influence of identified microbial communities on neurodevelopment is a critical next step (Walter et al., 2020). Subsequently, carefully designed clinical trials evaluating targeted interventions (e.g., probiotics or postbiotics aimed at promoting beneficial taxa like *Akkermansia muciniphila* (Plovier et al., 2017) will be necessary to determine if modulating this gut ecosystem trajectory can improve outcomes (Athalye-Jape and Patole, 2019). Our work defines the specific microbial and metabolic targets for such essential future studies.

In conclusion, this study identifies a gut ecosystem trajectory associated with high risk for NDI in preterm infants, with features detectable from the earliest days of life. The observed signatures, particularly the association between *Akkermansia* and glycerophospholipids, may represent candidate multi-omics biomarkers for early risk prediction, though this requires validation in independent cohorts. More broadly, these findings provide a rationale for further investigation into the relationship between the preterm gut microbiome and neurodevelopmental outcomes in future studies.

## 5 Conclusion

In summary, this prospective matched-cohort multi-omics study delineates a distinct developmental trajectory of the gut ecosystem in preterm infants classified as high-risk for neurodevelopmental impairment. This trajectory is characterized not by gross failure of microbial colonization, but by early commensal depletion (*Akkermansia muciniphila*), pathobiont expansion (*Klebsiella variicola*), a community-wide functional shift toward pro-inflammatory pathways, and failure to establish cooperative microbe-metabolite networks—features detectable from meconium and persisting through 3 months of age.

It is important to emphasize that these findings establish strong longitudinal associations between this gut ecosystem trajectory and neurodevelopmental risk, not causation. The detection of risk-associated features from the first days of life and their correlation with later neurobehavioral scores positions the gut microbiome as a promising early biomarker system. The observed multi-omics signatures, particularly the *Akkermansia*-glycerophospholipid module positively correlated with neurodevelopment, may represent candidate biomarkers for early risk stratification, though validation in independent cohorts is essential.

The primary translational value of this study lies in identifying a multi-omics signature associated with neurodevelopmental risk, which provides a theoretical foundation for future investigations—including experimental studies to establish causality and well-designed clinical trials to evaluate whether microbiome-targeted approaches could ultimately support neurodevelopment in this vulnerable population—thereby laying the groundwork for improving neurodevelopmental outcomes in preterm infants.

## Data Availability

The datasets presented in this study can be found in online repositories. The raw sequencing data are available in the NCBI SRA database under BioProject accession number PRJNA1428929 (https://dataview.ncbi.nlm.nih.gov/object/PRJNA1428929?reviewer=pjb5bk9qj4sucetr4nb0e2er0p). The metabolomics data are available in the MetaboLights database under accession number MTBLS13962 (https://www.ebi.ac.uk/metabolights/reviewerfa3c0502-83a7-4778-adde-8cdaa2ea5905).

## References

[B1] Arboleya S. Sánchez B. Milani C. Duranti S. Solís G. Fernández N. . (2015). Intestinal microbiota development in preterm neonates and effect of perinatal antibiotics. J. pediatrics. 166, 538–544. doi: 10.1016/j.jpeds.2014.09.041, PMID: 25444008

[B2] Athalye-Jape G. Patole S. (2019). Probiotics for preterm infants - time to end all controversies. Microbial Biotechnol. 12, 249–253. doi: 10.1111/1751-7915.13357, PMID: 30637944 PMC6389843

[B3] Behnsen J. Deriu E. Sassone-Corsi M. Raffatellu M. (2013). Probiotics: Properties, examples, and specific applications. Csh. Perspect. Med. 3, a10074. doi: 10.1101/cshperspect.a010074, PMID: 23457295 PMC3579206

[B4] Blencowe H. Cousens S. Oestergaard M. Z. Chou D. Moller A. Narwal R. . (2012). National, regional, and worldwide estimates of preterm birth rates in the year 2010 with time trends since 1990 for selected countries: A systematic analysis and implications. Lancet (London England). 379, 2162–2172. doi: 10.1016/S0140-6736(12)60820-4, PMID: 22682464

[B5] Bokobza C. Van Steenwinckel J. Mani S. Mezger V. Fleiss B. Gressens P. (2019). Neuroinflammation in preterm babies and autism spectrum disorders. Pediatr. Res. 85, 155–165. doi: 10.1038/s41390-018-0208-4, PMID: 30446768

[B6] Bokulich N. A. Chung J. Battaglia T. Henderson N. Jay M. Li H. . (2016). Antibiotics, birth mode, and diet shape microbiome maturation during early life. Sci. Transl. Med. 8, 343r–382r. doi: 10.1126/scitranslmed.aad7121, PMID: 27306664 PMC5308924

[B7] Borre Y. E. O’Keeffe G. W. Clarke G. Stanton C. Dinan T. G. Cryan J. F. (2014). Microbiota and neurodevelopmental windows: Implications for brain disorders. Trends Mol. Med. 20, 509–518. doi: 10.1016/j.molmed.2014.05.002, PMID: 24956966

[B9] Campbell S. K. Kolobe T. H. Osten E. T. Lenke M. Girolami G. L. (1995). Construct validity of the test of infant motor performance. Phys. Ther. 75, 585–596. doi: 10.1093/ptj/75.7.585, PMID: 7604077

[B8] Campbell S. K. Kolobe T. H. A. Wright B. D. Linacre J. M. (2002). Validity of the Test of Infant Motor Performance for prediction of 6-, 9- and 12-month scores on the Alberta Infant Motor Scale. Dev. Med. Child Neurol. 44, 263–272. doi: 10.1017/s0012162201002043, PMID: 11995895

[B10] Cani P. D. de Vos W. M. (2017). Next-generation beneficial microbes: the case of akkermansia muciniphila. Front. Microbiol. 8. doi: 10.3389/fmicb.2017.01765, PMID: 29018410 PMC5614963

[B11] Carlson A. L. Xia K. Azcarate-Peril M. A. Goldman B. D. Ahn M. Styner M. A. . (2018). Infant gut microbiome associated with cognitive?development. Biol. Psychiat. 83, 148–159. doi: 10.1016/j.biopsych.2017.06.021, PMID: 28793975 PMC5724966

[B12] Cervenka I. Agudelo L. Z. Ruas J. L. (2017). Kynurenines: Tryptophan’s metabolites in exercise, inflammation, and mental health. Sci. (New York N.Y.). 357, f9794. doi: 10.1126/science.aaf9794, PMID: 28751584

[B13] Chen X. Song W. Song Y. Cao H. Xu X. Li S. . (2023). Lipidomics reveal the cognitive improvement effects of Acer truncatum Bunge seed oil on hypoxic-ischemic encephalopathy rats. Food Funct. 14, 6610–6623. doi: 10.1039/d3fo01583a, PMID: 37395364

[B14] Chu D. M. Ma J. Prince A. L. Antony K. M. Seferovic M. D. Aagaard K. M. (2017). Maturation of the infant microbiome community structure and function across multiple body sites and in relation to mode of delivery. Nat. Med. 23, 314–326. doi: 10.1038/nm.4272, PMID: 28112736 PMC5345907

[B15] Cowan C. S. M. Dinan T. G. Cryan J. F. (2020). Annual Research Review: Critical windows - the microbiota-gut-brain axis in neurocognitive development. J. Child Psychol. psychiatry Allied disciplines. 61, 353–371. doi: 10.1111/jcpp.13156, PMID: 31773737

[B16] Cryan J. F. O’Riordan K. J. Cowan C. S. M. Sandhu K. V. Bastiaanssen T. F. S. Boehme M. . (2019). The microbiota-gut-brain axis. Physiol. Rev. 99, 1877–2013. doi: 10.1152/physrev.00018.2018, PMID: 31460832

[B17] Dao M. C. Everard A. Aron-Wisnewsky J. Sokolovska N. Prifti E. Verger E. O. . (2016). Akkermansia muciniphila and improved metabolic health during a dietary intervention in obesity: Relationship with gut microbiome richness and ecology. Gut. 65, 426–436. doi: 10.1136/gutjnl-2014-308778, PMID: 26100928

[B18] Derrien M. Belzer C. de Vos W. M. (2017). Akkermansia muciniphila and its role in regulating host functions. Microb. Pathogenesis. 106, 171–181. doi: 10.1016/j.micpath.2016.02.005, PMID: 26875998

[B19] Dodd D. Spitzer M. H. Van Treuren W. Merrill B. D. Hryckowian A. J. Higginbottom S. K. . (2017). A gut bacterial pathway metabolizes aromatic amino acids into nine circulating metabolites. Nature. 551, 648–652. doi: 10.1038/nature24661, PMID: 29168502 PMC5850949

[B20] Drobyshevsky A. Synowiec S. Goussakov I. Fabres R. Lu J. Caplan M. (2024). Intestinal microbiota modulates neuroinflammatory response and brain injury after neonatal hypoxia-ischemia. Gut Microbes 16, 2333808. doi: 10.1080/19490976.2024.2333808, PMID: 38533575 PMC10978030

[B21] Dzubak P. Hajduch M. Vydra D. Hustova A. Kvasnica M. Biedermann D. . (2006). Pharmacological activities of natural triterpenoids and their therapeutic implications. Nat. Prod. Rep. 23, 394–411. doi: 10.1039/b515312n, PMID: 16741586

[B22] Einspieler C. Prechtl H. F. R. (2005). Prechtl’s assessment of general movements: A diagnostic tool for the functional assessment of the young nervous system. Ment. Retard. Dev. Disabil. Res. Rev. 11, 61–67. doi: 10.1002/mrdd.20051, PMID: 15856440

[B23] Fu X. Du B. Chen P. Shama A. Chen B. Zhang X. . (2024). Exploring the impact of gut microbial metabolites on inactivated SARS-CoV-2 vaccine efficacy during pregnancy and mother-to-infant antibody transfer. Gut. 73, 1397–1400. doi: 10.1136/gutjnl-2023-330497, PMID: 37739779 PMC11287518

[B24] Gasparrini A. J. Wang B. Sun X. Kennedy E. A. Hernandez-Leyva A. Ndao I. M. . (2019). Persistent metagenomic signatures of early-life hospitalization and antibiotic treatment in the infant gut microbiota and resistome. Nat. Microbiol. 4, 2285–2297. doi: 10.1038/s41564-019-0550-2, PMID: 31501537 PMC6879825

[B25] Gensollen T. Iyer S. S. Kasper D. L. Blumberg R. S. (2016). How colonization by microbiota in early life shapes the immune system. Sci. (New York N.Y.). 352, 539–544. doi: 10.1126/science.aad9378, PMID: 27126036 PMC5050524

[B26] Gibson M. K. Wang B. Ahmadi S. Burnham C. D. Tarr P. I. Warner B. B. . (2016). Developmental dynamics of the preterm infant gut microbiota and antibiotic resistome. Nat. Microbiol. 1, 16024. doi: 10.1038/nmicrobiol.2016.24, PMID: 27572443 PMC5031140

[B27] Gorrie C. L. Mirceta M. Wick R. R. Edwards D. J. Thomson N. R. Strugnell R. A. . (2017). Gastrointestinal carriage is a major reservoir of klebsiella pneumoniae infection in intensive care patients. Clin. Infect. Dis. 65, 208–215. doi: 10.1093/cid/cix270, PMID: 28369261 PMC5850561

[B28] Gosalbes M. J. Llop S. Vallès Y. Moya A. Ballester F. Francino M. P. (2013). Meconium microbiota types dominated by lactic acid or enteric bacteria are differentially associated with maternal eczema and respiratory problems in infants. Clin. Exp. Allergy 43, 198–211. doi: 10.1111/cea.12063, PMID: 23331561

[B29] Grier A. Qiu X. Bandyopadhyay S. Holden-Wiltse J. Kessler H. A. Gill A. L. . (2017). Impact of prematurity and nutrition on the developing gut microbiome and preterm infant growth. Microbiome. 5, 158. doi: 10.1186/s40168-017-0377-0, PMID: 29228972 PMC5725645

[B30] Hadders-Algra M. Klip-Van Den Nieuwendijk A. Martijn A. van Eykern L. A. (1997). Assessment of general movements: Towards a better understanding of a sensitive method to evaluate brain function in young infants. Dev. Med. Child Neurol. 39, 88–98. doi: 10.1111/j.1469-8749.1997.tb07390.x, PMID: 9062423

[B31] Hamilton J. A. Hillard C. J. Spector A. A. Watkins P. A. (2007). Brain uptake and utilization of fatty acids, lipids and lipoproteins: Application to neurological disorders. J. Mol. neuroscience: MN. 33, 2–11. doi: 10.1007/s12031-007-0060-1, PMID: 17901539

[B32] Hermans E. C. van Gerven C. C. E. Johnsen L. Tungen J. R. E. Nijboer C. H. de Theije C. G. M. (2024). Dietary LPC-Bound n-3 LCPUFA Protects against Neonatal Brain Injury in Mice but Does Not Enhance Stem Cell Therapy. Nutrients. 16, 2252. doi: 10.3390/nu16142252, PMID: 39064695 PMC11279425

[B33] Hill C. J. Lynch D. B. Murphy K. Ulaszewska M. Jeffery I. B. O’Shea C. A. . (2017). Evolution of gut microbiota composition from birth to 24 weeks in the INFANTMET Cohort. Microbiome. 5, 4. doi: 10.1186/s40168-016-0213-y, PMID: 28095889 PMC5240274

[B34] Knights D. Ward T. L. McKinlay C. E. Miller H. Gonzalez A. McDonald D. . (2014). Rethinking “enterotypes. Cell Host Microbe 16, 433–437. doi: 10.1016/j.chom.2014.09.013, PMID: 25299329 PMC5558460

[B35] Lamari F. Rossignol F. Mitchell G. A. (2025). Glycerophospholipids: Roles in cell trafficking and associated inborn errors. J. Inherit. Metab. Dis. 48, e70019. doi: 10.1002/jimd.70019, PMID: 40101691 PMC11919462

[B36] Martin R. M. Bachman M. A. (2018). Colonization, infection, and the accessory genome of klebsiella pneumoniae. Front. Cell. Infect. Mi. 8. doi: 10.3389/fcimb.2018.00004, PMID: 29404282 PMC5786545

[B37] Masi A. C. Stewart C. J. (2019). The role of the preterm intestinal microbiome in sepsis and necrotising enterocolitis. Early Hum. Dev. 138, 104854. doi: 10.1016/j.earlhumdev.2019.104854, PMID: 31481262

[B38] Morais L. H. Schreiber H. L. T. Mazmanian S. K. (2021). The gut microbiota-brain axis in behaviour and brain disorders. Nat. Rev. Microbiol. 19, 241–255. doi: 10.1038/s41579-020-00460-0, PMID: 33093662

[B39] Ottman N. Geerlings S. Y. Aalvink S. de Vos W. M. Belzer C. (2017). Action and function of Akkermansia muciniphila in microbiome ecology, health and disease. Best Pract. Res. Clin. gastroenterology. 31, 637–642. doi: 10.1016/j.bpg.2017.10.001, PMID: 29566906

[B40] Pammi M. Cope J. Tarr P. I. Warner B. B. Morrow A. L. Mai V. . (2017). Intestinal dysbiosis in preterm infants preceding necrotizing enterocolitis: A systematic review and meta-analysis. Microbiome. 5, 31. doi: 10.1186/s40168-017-0248-8, PMID: 28274256 PMC5343300

[B41] Plovier H. Everard A. Druart C. Depommier C. Van Hul M. Geurts L. . (2017). A purified membrane protein from Akkermansia muciniphila or the pasteurized bacterium improves metabolism in obese and diabetic mice. Nat. Med. 23, 107–113. doi: 10.1038/nm.4236, PMID: 27892954

[B42] Rooks M. G. Garrett W. S. (2016). Gut microbiota, metabolites and host immunity. Nat. Rev. Immunol. 16, 341–352. doi: 10.1038/nri.2016.42, PMID: 27231050 PMC5541232

[B44] Sasabe J. Miyoshi Y. Rakoff-Nahoum S. Zhang T. Mita M. Davis B. M. . (2016). Interplay between microbial d-amino acids and host d-amino acid oxidase modifies murine mucosal defence and gut microbiota. Nat. Microbiol. 1, 16125. doi: 10.1038/nmicrobiol.2016.125, PMID: 27670111 PMC5074547

[B45] Schwarzer M. Makki K. Storelli G. Machuca-Gayet I. Srutkova D. Hermanova P. . (2016). Lactobacillus plantarum strain maintains growth of infant mice during chronic undernutrition. Sci. (New York N.Y.). 351, 854–857. doi: 10.1126/science.aad8588, PMID: 26912894

[B46] Seesahai J. Luther M. Church P. T. Maddalena P. Asztalos E. Rotter T. . (2021). The assessment of general movements in term and late-preterm infants diagnosed with neonatal encephalopathy, as a predictive tool of cerebral palsy by 2?years of age-a scoping review. Systematic Rev. 10, 226. doi: 10.1186/s13643-021-01765-8, PMID: 34384482 PMC8359053

[B47] Sharon G. Sampson T. R. Geschwind D. H. Mazmanian S. K. (2016). The central nervous system and the gut microbiome. Cell. 167, 915–932. doi: 10.1016/j.cell.2016.10.027, PMID: 27814521 PMC5127403

[B48] Silva Y. P. Bernardi A. Frozza R. L. (2020). The role of Short-Chain fatty acids from gut microbiota in Gut-Brain communication. Front. endocrinology. 11. doi: 10.3389/fendo.2020.00025, PMID: 32082260 PMC7005631

[B49] Strunk T. Inder T. Wang X. Burgner D. Mallard C. Levy O. (2014). Infection-induced inflammation and cerebral injury in preterm infants. Lancet Infect. diseases. 14, 751–762. doi: 10.1016/S1473-3099(14)70710-8, PMID: 24877996 PMC4125363

[B50] Thompson A. L. Monteagudo-Mera A. Cadenas M. B. Lampl M. L. Azcarate-Peril M. A. (2015). Milk- and solid-feeding practices and daycare attendance are associated with differences in bacterial diversity, predominant communities, and metabolic and immune function of the infant gut microbiome. Front. Cell. Infect. Mi. 5. doi: 10.3389/fcimb.2015.00003, PMID: 25705611 PMC4318912

[B51] Valles-Colomer M. Falony G. Darzi Y. Tigchelaar E. F. Wang J. Tito R. Y. . (2019). The neuroactive potential of the human gut microbiota in quality of life and depression. Nat. Microbiol. 4, 623–632. doi: 10.1038/s41564-018-0337-x, PMID: 30718848

[B52] Walter J. Armet A. M. Finlay B. B. Shanahan F. (2020). Establishing or exaggerating causality for the gut microbiome: lessons from human microbiota-associated rodents. Cell. 180, 221–232. doi: 10.1016/j.cell.2019.12.025, PMID: 31978342

[B53] Wang X. Fan Y. Dong Y. Zhang Y. Tan X. Gai Z. . (2025). Strain-Specific safety evaluation of akkermansia muciniphila akk11: Comprehensive genotypic, phenotypic, and toxicological assessment. Food Sci. Nutr. 13, e71154. doi: 10.1002/fsn3.71154, PMID: 41200210 PMC12586882

[B54] Wang Y. Zhu J. Zou N. Zhang L. Wang Y. Zhang M. . (2023). Pathogenesis from the microbial-gut-brain axis in white matter injury in preterm infants: A review. Front. Integr. Neurosci. 17. doi: 10.3389/fnint.2023.1051689, PMID: 37006416 PMC10060642

[B55] Xiao J. (2024). Role of the gut Microbiota-Brain axis in brain damage in preterm infants. ACS Pharmacol. Trans. science. 7, 1197–1204. doi: 10.1021/acsptsci.3c00369, PMID: 38751622 PMC11091980

[B56] Xue C. Lv H. Li Y. Dong N. Wang Y. Zhou J. . (2022). Oleanolic acid reshapes the gut microbiota and alters immune-related gene expression of intestinal epithelial cells. J. Sci. Food Agr. 102, 764–773. doi: 10.1002/jsfa.11410, PMID: 34227118

[B58] Yassour M. Vatanen T. Siljander H. Hämäläinen A. M. Härkönen T. Ryhänen S. J. . (2016). Natural history of the infant gut microbiome and impact of antibiotic treatment on bacterial strain diversity and stability. Sci. Transl. Med. 8, 343r–381r. doi: 10.1126/scitranslmed.aad0917, PMID: 27306663 PMC5032909

[B59] Yee A. L. Miller E. Dishaw L. J. Gordon J. M. Ji M. Dutra S. . (2019). Longitudinal microbiome composition and stability correlate with increased weight and length of Very-Low-Birth-Weight infants. mSystems. 4, e218–e229. doi: 10.1128/mSystems.00229-18, PMID: 30834328 PMC6392092

[B60] Zhang T. Yin Y. Xia X. Que X. Liu X. Zhao G. . (2026). Regulation of synaptic function and lipid metabolism. Neural Regen. Res. 21, 1037–1057. doi: 10.4103/NRR.NRR-D-24-01412, PMID: 40313084 PMC12296456

